# Asthma and Air Pollution: What’s Happening in NIEHS Extramural Research

**Published:** 2005-01

**Authors:** Sally S. Tinkle

**Affiliations:** tinkle@niehs.nih.gov

Asthma is a chronic inflammatory disease with symptoms including reversible airway constriction, chest tightness, cough, and wheezing. The incidence of asthma is increasing and accounts for nearly 500,000 hospitalizations, 2 million emergency department visits, and 5,000 deaths annually in the United States. Asthma develops most commonly in children, although recent data suggest an increase in new cases among adults and the elderly.

An individual’s risk for developing asthma is defined by a complex interaction of environmental exposures and hereditary factors. Risk factors include atopy or a predisposition to a Th2 immune response, diminished childhood microbe exposure, age at time of critical exposure, obesity, urbanization, and low socioeconomic status. In addition, numerous epidemiological studies have linked air pollution to exacerbation of acute asthma, increased use of asthma medication, increased school and work absence, and increased hospitalization. The NIEHS, recognizing these links and the persistence and continuing increase in air pollution globally, supports numerous research investigations that may provide keys to improved prevention and clinical management of asthma.

Toxicological research has characterized several components of air pollution, including particulate matter (PM), gaseous elements such as ozone, microbial products including endotoxin, heavy metals, and indoor and outdoor allergens such as house dust mite allergen and ragweed. Current NIEHS-sponsored extramural research targets pulmonary injury and dysfunction consequent to these exposures.

For example, researchers are examining the cellular and molecular pathways involved in oxidative stress induced by organic and metal-containing PM. Oxidative stress is a component of the inflammatory response and of airway hyperreactivity and asthma exacerbation. Other investigators are determining the cellular mechanisms though which diesel exhaust particles act as an adjuvant for common environmental allergens and contribute to the increased incidence of allergies and allergic asthma. Several laboratories are exploring outcomes of ozone exposure including neutrophilic inflammation, cytokine production, and impaired pulmonary function, while others are testing the impact of perinatal ozone exposure, in combination with house dust mite exposure, on lung maturation and childhood asthma.

Genetics research focuses on candidate genes whose expression is altered by environmental exposures that contribute to asthma development. Current studies are investigating known polymorphisms in the pulmonary surfactant proteins important to host defense and in the Th2 cytokines, such as IL-13, that drive the asthma response. Genomics studies are also in progress to identify new asthma susceptibility genes and polymorphic markers of disease.

On 18–19 October 2004, the NIEHS and the U.S. Environmental Protection Agency cosponsored the workshop Environmental Influences on the Induction and Incidence of Asthma. Participants reviewed the current scientific evidence on factors that contribute to the induction and increased incidence of asthma, and small interdisciplinary discussion groups identified research questions critical to improved understanding of the induction of asthma and clinical management. The workshop’s conclusions highlighted the need to identify the critical windows of perinatal lung development and to understand how environmental exposures during these developmental windows leads to asthma. [For more on this workshop, see “Environmental Roots of Asthma,” p. A32–A33 this issue.]

